# Implant—Abutment Misfit after Cyclic Loading: An In Vitro Experimental Study

**DOI:** 10.3390/ma15155341

**Published:** 2022-08-03

**Authors:** John Eversong Lucena de Vasconcelos, Jefferson David Melo de Matos, Daher Antonio Queiroz, Guilherme da Rocha Scalzer Lopes, Bruna Caroline Gonçalves Vasconcelos de Lacerda, Marco Antonio Bottino, Cecilia Pedroso Turssi, Roberta Tarkany Basting, Flávia Lucisano Botelho do Amaral, Fabiana Mantovani Gomes França

**Affiliations:** 1 Department of Implantology, College of Dentistry CECAPE, Juazeiro do Norte 63024-015, CE, Brazil; johnelvasconcelos@yahoo.com.br (J.E.L.d.V.); or melodematos.j@ufl.edu (J.D.M.d.M.); brunacgvasconcelos@hotmail.com (B.C.G.V.d.L.); 2 Dental Research Center, São Leopoldo Mandic Institute, Campinas 13045-755, SP, Brazil; cecilia.turssi@gmail.com (C.P.T.); rbasting@yahoo.com (R.T.B.); flbamaral@gmail.com (F.L.B.d.A.); biagomes@yahoo.com (F.M.G.F.); 3 Center for Dental Biomaterials, Department of Restorative Dental Sciences, University of Florida (UF Health), Gainesville, FL 32611, USA; 4 Department of Biomaterials, Dental Materials, and Prosthodontics, Institute of Science and Technology, São Paulo State University (Unesp), São José dos Campos 05508-070, SP, Brazil; guilherme.scalzer@unesp.br (G.d.R.S.L.); marco.bottino@unesp.br (M.A.B.); 5 The University of Texas Health Science Center, Department of Restorative Dentistry & Prosthodontics, Houston (UTHealth) School of Dentistry, Houston, TX 77054, USA

**Keywords:** implant-supported fixed dental prostheses, dental implants, biomechanics

## Abstract

This study aimed to evaluate the influence of thermomechanical cycling (TMC) and type of abutment on the misfit and compressive strength of the implant–abutment interface. Forty 3.75-mm external hexagon implants with 25° angled abutments were divided into four groups (N = 10). Group A: overcast plus TMC; Group B: overcast without TMC; Group C: completely cast plus TMC; Group D: completely cast without TMC. Abutments were fixed to the implants with 32-Ncm torque, and groups A and C specimens were cyclically loaded at 80 N with 2 Hz for 1 million cycles. The misfit on the implant–abutment interface was evaluated by optical microscope (100×) and the compressive strength test was performed in a universal test machine. For statistical analysis, a two-way ANOVA and post hoc Tukey test were used. There was no difference in misfit presented by all the abutments in the absence of TMC (*p* > 0.05). When TMC was performed, the completely cast abutments showed greater misfit than overcast ones (*p* = 0.001). Regarding compressive strength, irrespective of TMC performed, the overcast abutments showed higher compressive strength values than completely cast abutments (*p* = 0.003). Moreover, disregarding the type of abutment used, the absence of TMC provided higher compressive strength values (*p* < 0.001). It was concluded that thermomechanical cyclic loading aggravated the misfit, especially in completely cast abutments, regardless of material or fabrication technique, and reduced the compressive strength of the two types of abutments tested.

## 1. Introduction

High success rates have been documented in rehabilitation with osseointegrated implants. Nevertheless, the longevity of implant-supported restorations depends on biomechanical factors, such as accuracy among the components to ensure stability, resistance, and esthetic results [1,2]. Among the prosthetic complications, the most recurrent is prosthetic screw loosening or fracture. Loosening of the screw is a well-documented issue [3,4,5,6,7,8,9,10] in external hexagon implants, due to the small height of the hexagon connection, which provides lower stability against lateral loads, and thus the screw is vulnerable to shear loads [11,12,13,14,15].

The different abutment types and casting technics may influence the implant/abutment adaptation [16,17]. Castable abutments may or may not have a metal base in the region of adaptation to the implant and the casting may be made by induction or by the lost wax technique [2,15,17,18,19]. These factors may influence the misfit of the implant/abutment assembly and, consequently, affect the mechanical performance of the prosthetic screws. The casting procedures produce irregularities and roughness on the surfaces of prosthetic components, which may change the mechanical performance and structural properties of the surfaces that are in contact during the process of tightening the screws [20].

The passive fit of restorations is essential for successful rehabilitation with implants [18], because lack of passive fit in implant-supported prostheses induces deleterious forces on the implants and their associated components, and may lead to failure of the prosthesis due to fatigue and/or loss of osseointegration [21,22]. A microcap equal to or greater than 30 µm may be considered doubtful or clinically unacceptable [23]. The micro gap is influenced by various factors, including the precision of the milling method [16]; correct torque of the prosthetic screw [24,25]; casting technique [26]; and the type of metal used in the process [2,15,16].

Biomechanical complications are known to occur more frequently when lateral or oblique forces are present [10,27]. In their turn, lateral forces seem to be more deleterious in angled abutments [28,29]. In effect, transversal forces are considered more harmful because of the lower resistance of the components to shear stress or forces, and flexure caused by the height of the crown [30,31]. Excessive occlusal force may lead to screw loosening and create a gap [32]. Therefore, occlusal fatigue and non-passivity between the implant–abutment components may cause frequent prosthetic screw fractures or loosening and, also, implant fractures [6].

The fracture resistance test is an important method for evaluating the maximum load supported by the implant [33], enabling a comparative analysis to be made of the resistance between different types of implants and components. This is because analysis of the samples after tests may point out different conditions of failures, and the type and location of these failures may bring to light important information about the clinical behavior and future of the implants [12,34]. Several studies have reported that cycling increased the misfit of machined abutments [8,35,36,37] and others have recorded that the compressive strength of the implant/abutment assembly is also influenced by cycling loading [3,17,38].

Therefore, this study aimed to evaluate the influence of thermomechanical fatigue cycles and different types of the castable abutment on the adaptation and compressive strength of the implant–abutment interface. The null hypothesis tested was that mechanical cycling and the presence of the metal base on the castable abutment would not influence the adaptation and compressive strength of the implant/abutment interface.

## 2. Materials and Methods

**Figure 1:**
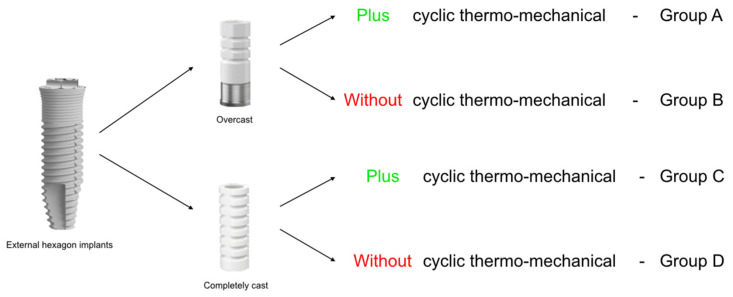
Experimental groups with different abutments.

A total of 40 external hexagon implants 3.75 × 13 mm type Branemark (Zimmer Biomet, Dover, OH, USA) with a 4.1-mm platform, 20 external hexagonal metal base compatible abutments (Zimmer Biomet, Dover, OH, USA), and 20 external hexagonal plastic compatible abutments (Zimmer Biomet, Dover, OH, USA) were assigned to 4 groups of 10 specimens, according to the type of abutment, and submitted to thermomechanical cycling (TMC): overcast/TMC group (A), overcast/non-TMC group (B), completely cast/TMC group (C), completely cast/non-TMC group (D) (Table 1 and Figure 1).

To standardize the prosthetic abutments, a master 25° angle abutment was made with auto-polymerizing resin (Pattern Resin LS; GC Pattern Resin LS, Tokyo, Japan). A silicone mold (Extrude XP putty, Kerr Corp., Orange, CA, USA) was fabricated to assist in waxing the abutments. A total of 20 external hexagonal metal base compatible abutments and 20 anti-rotational external hexagonal plastic compatible abutments were attached to the implant analog, and wax was applied to build the abutment to complete contour with a silicone mold. A total of 20 waxed pre-machined anti-rotational custom dental implant abutments in cobalt–chromium abutments and 20 waxed anti-rotational castable custom dental implant abutments were cast in Ni–Cr alloy by induction and lost wax technique, respectively. All waxing and casting were completed by 1 investigator (J.E.L.d.T.) for consistency. The wax patterns were individually invested by using phosphate-bonded investment (Ceramigold; Whip Mix Corp., Louisville, KY, USA) and cast with nickel–chromium alloy (Talmax, Curitiba, Paraná, Brazil). After casting, the specimens were bench-cooled and devested by airborne particle abrasion with 100-mm aluminum oxide and 6-MPa pressure. No further finishing or polishing was performed.

**Figure 2:**
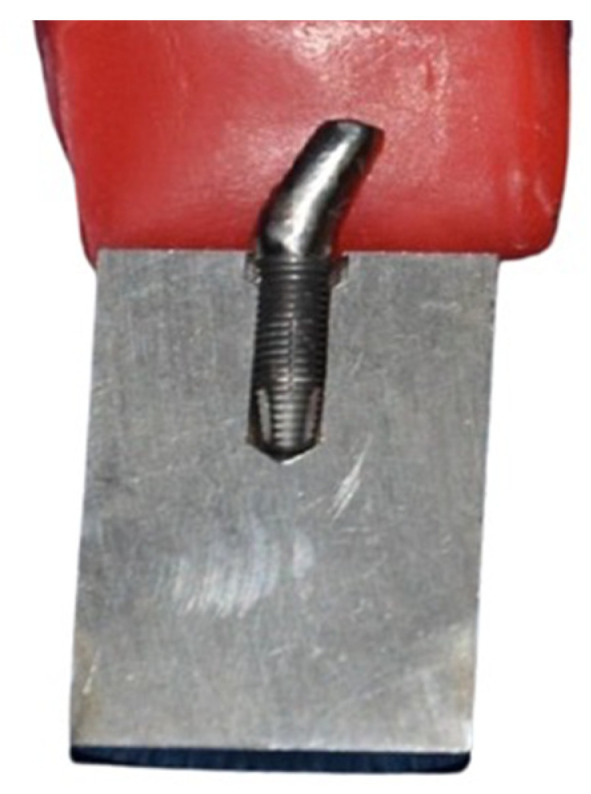
Sagittal view of the implant–abutment assembly.

All abutments were attached to the implants with titanium retaining screws (Zimmer Biomet, Dover, OH, USA) to the manufacturer’s recommended torque of 32 Ncm by using an analog torque wrench (BTG60CN-S model; Tohnichi Mfg. Co. Ltd., Tokyo, Japan). The assembly was then mounted in a testing apparatus in the predetermined position. To avoid loss of preload due to the effect of relaxation or initial tightening of the screw, the torque was applied twice, with an interval of 10 min (Figure 2) [39,40]. 

### 2.1. Thermomechanical Cycling

The cyclic load tests were performed using a dynamic test with a machine simulating fatigue by thermal cycling aging (Equip, São Carlos, SP, Brazil), and 1 million cycles per sample were applied with 80 N loading, at a speed/frequency of 2 Hz, the equivalent of approximately 12 months of function [18]. After the fatigue test, the prosthetic implant/abutment assembly was removed from the embedment with the aid of heated water.

### 2.2. Evaluation of the Implant/Abutment Interface Misfit

All the samples were positioned in the digital stereomicroscope (Discovery V20, CarlZeiss, Jena Thuringia, Germany), and the buccal, lingual, mesial, and distal surfaces of each specimen were evaluated on the precision of margins at the implant–abutment interface at 100x magnification.

### 2.3. Compressive Load Test

**Figure 3:**
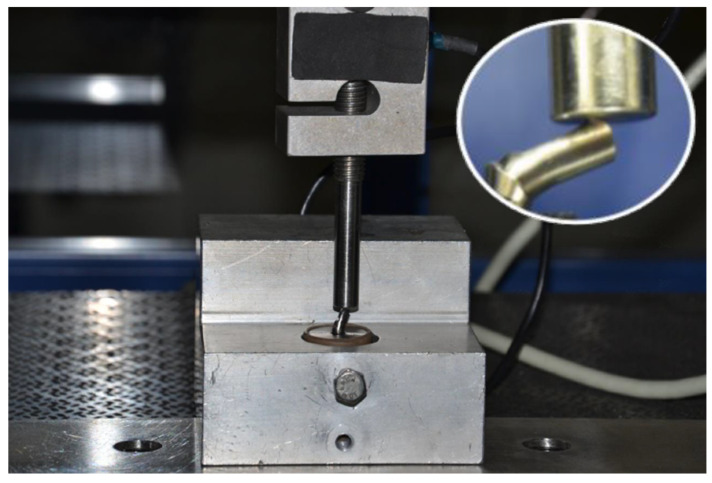
Compressive load test with Trd 26 load cell.

Specimens were placed into a mechanical testing machine (EMIC DL2000 Ind. e Com. LTDA, São José dos Pinhais/PR, Brazil) to measure the resistance to compressive forces with Trd 26 load cell until some of the components and screws fractured, loosened, or underwent deformation. The implant/abutment assembly was submitted to compressive loading of 2000 kg at a speed of 1.0 mm/min (Figure 3).

After the test, the assembly was removed from the device and the fracture pattern was examined, under a 30x magnification, and classified into: implant fracture; screw fracture—first thread; screw fracture—central threads; screw fracture—most apical thread; screw plastic deformation (no fracture); implant fracture; abutment misfit; shearing of the abutment; implant platform deformation; implant external hexagon deformation; abutment internal hexagon deformation; or there were no visible deformations.

### 2.4. Scanning Electron Microscopy

After the compressive strength test, the failure modes were illustrated by scanning electron microscopy (JEOL 5900LV, Japan Electro-Optics Labs, Tokyo, Japan) with a focus on the nature of the implant–abutment interface, generating digital microphotographs of the magnified areas.

### 2.5. Statistical Analysis

The misfit and resistance to fracture were analyzed with a repeated-measure, 2-way ANOVA and post hoc Tukey test were used (SPSS 20, SPSS Inc., Chicago, IL, USA), and the data for correlation between misfit and screw fracture were evaluated by the Pearson test (α = 0.05).

## 3. Results

For implant/abutment misfit data, the two-way analysis of variance showed a significant effect of the interaction between the studied variables (*p* = 0.001). Table 2 shows the mean values and Tukey test (95%) of the marginal misfits of the specimens. No statistically significant difference in marginal misfit was found between A (9.1 ± 3.6 µm) and C (6.9 ± 1.9 µm) groups in the absence of TMC. When TMC was performed, a statistically significant difference was found, and a higher marginal misfit was presented by the D (17.1 ± 4.3 µm) than the C group (6.7 ± 2.5 µm).

No statistically significant difference in marginal misfit was found between overcast abutments (A and C) groups, whether or not thermomechanical cycling was performed.

For implant/abutment assembly fracture resistance data, the two-way analysis of variance showed no significant effect of the interaction between the studied variables (*p* = 0.932). The authors found that, regardless of whether or not thermomechanical cycling was performed, overcast abutments presented significantly higher compressive strength values than those verified for the complete cast abutments (*p* = 0.003). Furthermore, it was shown that, without considering the abutment type used, the absence of thermomechanical cycling provided statistically higher compressive strength values (*p* < 0.001), as may be noted in Table 2.

**Figure 4:**
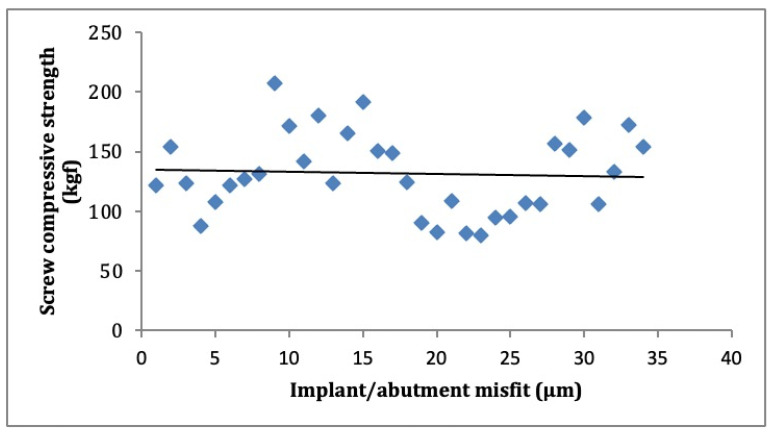
Dispersion diagram of implant/abutment misfit and compressive strength of connector screws.

The Pearson test demonstrated a negative but weak correlation between the implant/abutment interface misfit and resistance to compression values of the implant/abutment assembly (*p* = 0.019; r^2^ = −0.383), as may be noted in Figure 4.

The specimens were evaluated in failure mode and classified into one of the types of fracture, as described in Table 3. Plastic deformation of the screw (no fracture) and abutment misfit represented 90% of the failures found in both types of abutments not submitted to thermomechanical cycling and in the completely cast abutments/TMC. In the presence of TMC, 100% of the overcast abutment screws presented plastic deformation (no fracture).

Abutment misfit was verified in 90% of the specimens in the A, B, and C groups. All specimens in the D group revealed abutment misfit.

Deformation of the implant platform occurred in 100% of the overcast abutments when these were subjected to thermomechanical cycling, whereas, in the absence of TMC, 80% of the overcast abutments demonstrated deformation. In the completely cast abutments, plastic platform deformation was present in 80% when TMC was absent, and in 70% when TMC was performed.

External hexagon implant deformation affected 60% of the complete cast abutments submitted to TMC, and half of the specimens in the B group. In abutments not submitted to TMC, the failures due to deformation of the implant external hexagon occurred in a lower proportion in the overcast abutments (40%) and completely cast abutments (30%).

**Figure 5:**
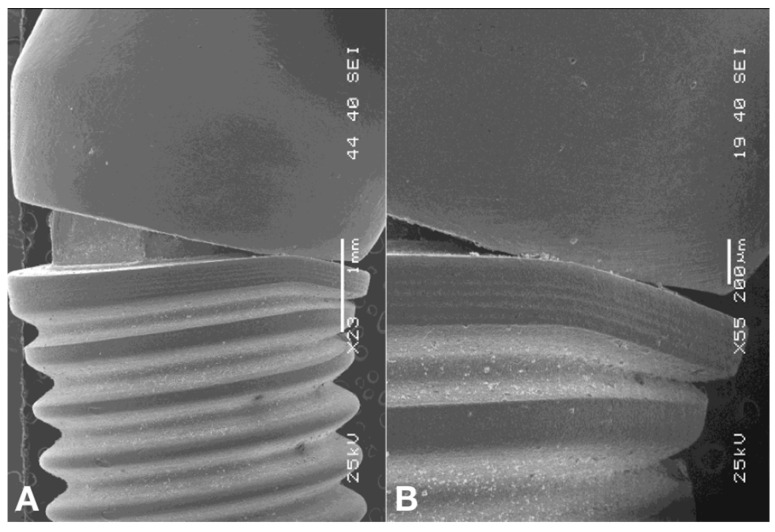
(**A**) On the angulation side of the abutment, the edge of the implant experienced plastic deformations characterized by crushing and consequent increase in marginal misfit. (**B**) Nevertheless, depending on the area measured, the gap diminished by compression of the abutment edge against the implant edge.

**Figure 6:**
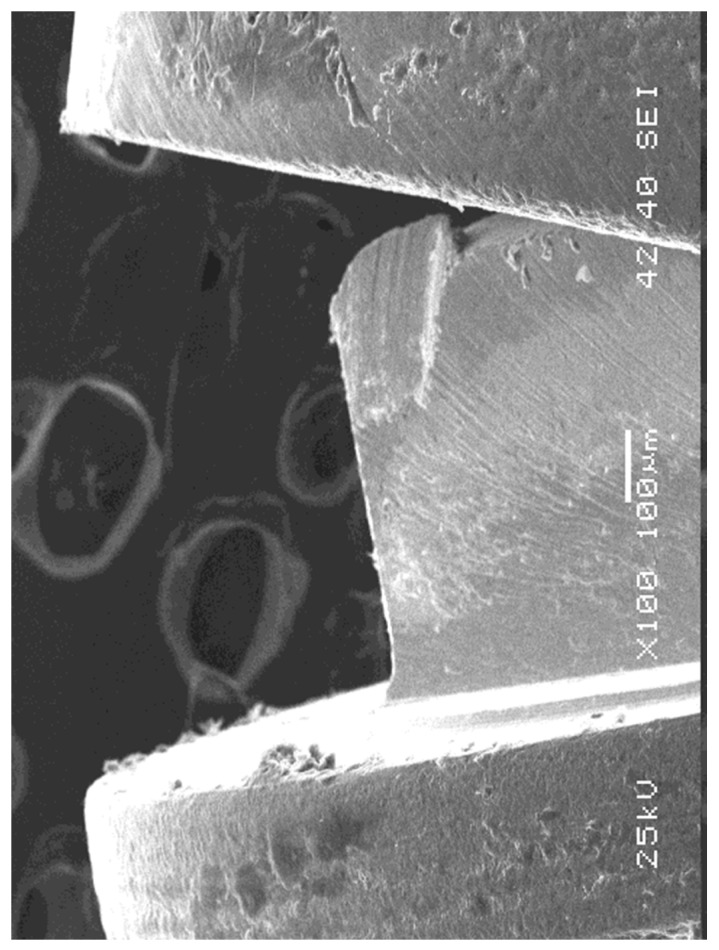
On the side opposite the angulation of the abutment, the gap opened considerably, and the hexagon suffered crushing or scratching, at times with the loss of substance.

The SEM images demonstrated that, on the abutment angulation side, the implant base experienced plastic deformations characterized by crushing and marginal misfit enlargement. Nevertheless, depending on the area measured, the gap diminished by compression of the abutment edge against the implant edge (Figure 5A,B). Meanwhile, on the opposite side, the marginal misfit opened considerably and the external hexagon was crushed and scratched, with loss of substance, by abutment elevation at the implant edge (Figure 6).

## 4. Discussion

The vulnerability of the implant/abutment assembly to the masticatory loads exposes all the components to fatigue and plastic deformations [12,41] and may result in technical or mechanical complications associated with the restorations of single implants and implant-supported partial dental prostheses, such as screws loosening [5,6,7,8,9], wear [17], fracture of the screw and abutment, chipping or fracture of the lining material, and de-cementation [10]. These failures may be associated with the prosthetic connection and increased marginal misfit at the implant/abutment interface [11] and occlusal overload.

In this context, the present study used a screw-retained single implant, since this situation is more susceptible to mechanical failure than a fixed partial denture [42]. Moreover, this study investigated completely cast and overcast abutments based on the fact that the overcast has been reported to have superior mechanical behavior [2,18,19]. The materials used for casting include gold (Au), palladium–silver alloys, commercially pure titanium (Ti), cobalt–chrome (Co–Cr), nickel–chrome, and Ni–CR–Ti. In comparison with gold-based alloys, Co–Cr alloys have a high modulus of elasticity and significantly lower cost. They also have good resistance to corrosion and biocompatibility [16]. However, in pre-machined parts, there is the possibility of distortion of components caused by the casting procedure or during the porcelain firing process or a combination of the two procedures [17,20]. The Co–Cr overcast abutments, on the capacity of sealing the implant–abutment connection, were evaluated by Ramos et al. (2014) [43], who related microbial microleakage observed for all abutments studied, regardless of metal base abutments.

In this study, the specimens consisted of 25° angulated abutments that were obtained by casting abutments. The angulated abutment, prefabricated or waxed and cast in a laboratory from a burnout abutment, may be indicated for multiple restoration situations, correction of parallelism between implants, and customized single restorations. The possibility of correcting implant positioning with an angulated abutment requires laboratory steps that may cause abutment misfit, resulting in screw loosening and/or fracture [27].

Misfit and micromotion of the implant–abutment assembly may be the causes of bone resorption around the neck of the dental implant [22]. The implant–abutment interface is also a significant factor in stress conduction to the surrounded bone. Misfits may be classified as vertical, horizontal, angular, and rotational. Vertical and rotational misfits have been widely discussed because they are related to the most common mechanical problems reported. Horizontal misfit represents the under- and over-contour between the abutment and implant. Angular and vertical misfits are similar, since both are characterized by gaps at the implant–abutment interface, but the angular type, as the very name suggests, exhibits a misfit at an angle [18].

The aging process simulated was used to verify its influence on implant/abutment interface misfit by simulating masticatory loads [8,37]. Thermocycler load, also known as thermomechanical cycling, is characterized by the application of a previously defined load for a certain number of cycles at a certain frequency [33]. In this study, 1 million cycles of 80 N at 2 Hz were used to simulate one year of mastication, a period also adopted by Assunção (2011a) [18]. Despite their inherent limitations, mechanical fatigue tests have found backing in evaluating the performance of the implant–abutment assembly, making it possible to evaluate and compare the resistance between different types of implants and components, the failure modes, and where they are situated, thus contributing to the establishment of lasting and reliable implant dentistry [12].

Considering the factor misfit in this study, the authors observed an influence of thermomechanical cycling and the different abutments on the measurements of the implant–abutment interface, so the null hypothesis was rejected. Thermomechanical cycling increased the misfit of the complete cast abutment. This may have occurred as a result of processing errors during investing, casting, and airborne-particle abrasion. Therefore, contraction of the wax, expansion of the plasters and linings, and contraction of the resins and metals are factors that may contribute to the deficient stability of the implant–abutment assembly. Conversely, the overcast abutments, in which the adaptation accuracy is derived from the computed milling process, are, therefore, minimal gaps resulting from casting. This inadequate fitting may influence the success of implant-supported dental prostheses, since they largely depend on the passivity achieved and the patterns of stress distribution. Therefore, a certain level of misfit on the prosthetic crown may generate mechanical complications and affect longevity [18] by presenting components of stress and, consequently, result in failure, fracture of the implant, screw loosening, or microfracture of the bone, and bone loss [25,44]. The passive fit of an implant-supported prosthetic structure is defined as circumferential, simultaneous, stress-free contact at the implant–abutment interface before functional loading [2].

Component precision has been related as a factor that may change the preload and stability of the prosthetic connection [20]. Mechanical cycling results in internal micromovements between the implant–abutment assembly. These micro-movements, which may be horizontal and/or vertical, culminate in wear of the contact surfaces [17,36], resulting in an increased gap in the completely cast group. To Farina (2014) [25], implant-supported dental prostheses do not provide perfect adaptation and, therefore, residual static stresses are created. The magnitude of stares depends on the quantity of misfit, which suggests that these residual static stresses may change the behavior of the screw-multiple abutment prostheses stability when compared with single-unit implant-supported dental prostheses.

Different methodologies have been used to evaluate the misfit at the implant–abutment connection. Kahramanoglu et al. (2013) [45] used a light microscope at 48x magnification. In this study, to illustrate the interface and failure mode, SEM was used [17,22,38,40,42,46,47].

As regards the factor resistance to compression, the null hypothesis was also rejected because thermomechanical cycling and abutment type influenced the performance of the groups. The authors observed that the presence of the Cr–Co–Mo overcast abutments guaranteed greater resistance to compression than was shown by the completely cast abutments. The higher resistance of the abutment with metal base shown in this study was probably due to the greater precision of adaptation between the parts, since the metal base supplied by the manufacturer turns the abutment interface independent of the inconsistencies inherent to casting, which contradicts what Queiroz et al. (2020) [17] stated in their study. As the mechanical cycling procedure tends to destabilize the internal connection between the components, this justifies the reduction in the resistance to compression of the specimens shown in this study.

The negative but weak correlation between the implant–abutment misfit values and compression strength of the implant–abutment assembly demonstrated that the greater the resistance to compression, the lower the interface misfit, confirming the findings of Aguirrebeitia et al. (2012) [48]. Some scratches were observed on the vertical extension of the hexagon in some implants. In others, there was the rounding of the hexagon, giving way to a circular section, corroborating the findings of Butignon et al. (2013) [3] and Khraisat (2013) [49].

None of the samples of this study presented fractures of the screw, in disagreement with others [50,51], who showed fractures of the screws occurred in 100% of the cases. This is justified because, according to the manufacturer of the system used in this study, its connector screws are made of Grade 5 Titanium alloy (TiAI6V4; TAV: 90% titanium; 6% aluminum; 4% vanadium), with aluminum and vanadium being elements that give the screw greater flexural and bending capacity, without fracturing. Titanium alloy screws are more resistant to failures than those made of commercially pure titanium [52].

In the abutment, failure occurred as the result of a moment of flexure, which led to permanent deformation of the abutment, in agreement with the study of Saninino et al. (2013) [12]. However, no deformation was observed in the internal hexagon of the abutment, whose base was shown to be very resistant to plastic deformations, a result that disagrees with the study of Butignon et al. (2013) [3], who showed plastic deformations in the internal hexagon of the abutment in the part related to the external hexagon of the implant.

Therefore, considering the cyclic fatigue present in the oral cavity, the authors inferred that the overcast abutments may be more resistant and maintain a smaller misfit at the implant–abutment interface compared with completely cast abutments.

## 5. Conclusions

Based on the findings of this in vitro study, the following conclusions were drawn:

- Mechanical cycling could increase the misfit at the interface for the complete cast angled abutments;
- Mechanical cycling could reduce the compressive strength for the overcast and completely cast angled abutments;
- Independent of cycling, overcast abutment showed better mechanical behavior than completely cast abutment.

## Figures and Tables

**Table 1:** Experimental groups.

Groups	N	4.1 Platform Dental Implant (mm)	Abutment	Used Material	Method	Submitted to Thermomechanical Cycling
A	10	3.75 × 13 mm P-I Branemark, Zimmer Holdings^®^	anti-rotational Co-Cr-Mo custom dental implant abutment	Ni–-Cr alloy	Induction technic	Yes
B	10	3.75 × 13 mm P-I Branemark, Zimmer Holdings^®^	anti-rotational Co-Cr-Mo custom dental implant abutment	Ni–Cr alloy	Induction technic	No
C	10	3.75 × 13 mm P-I Branemark, Zimmer Holdings^®^	anti-rotational custom dental implant abutment	Ni–Cr alloy	Conventional (lost wax)	Yes
D	10	3.75 × 13 mm P-I Branemark, Zimmer Holdings^®^	anti-rotational custom dental implant abutment	Ni–Cr alloy	Conventional (lost wax)	No

**Table 2:** Means and standard deviation of implant/abutment misfit and compressive strength of prosthetic screws, according to the type of abutment and presence of mechanical cycling.

UCLA Abutment	Implant/Abutment Misfit (µm)		Compressive Strength (Kgf)	
	without Cycling	Plus Cycling	without Cycling	Plus Cycling
Overcast	9.1 (3.6) Aa	6.7 (2.5) Aa	160.4 (27.7) Aa	122.8 (17.1) Ab
Completely cast	6.9 (1.9) Aa	17.1 (4.3) Bb	137.2 (30.6) Ba	98.3 (17.5) Bb

Legend: different letters indicate *p* < 0.05.

**Table 3:** Relative frequency (%) of failure modes observed in the implant/abutment according to the type of abutment and presence of mechanical cycle.

Failure Mode	Cr–Co–Mo Metal Strap		Calcinable Plastic	
	without Cycling	Plus Cycling	without Cycling	Plus Cycling
Implant Fracture	0%	0%	0%	0%
Screw fracture—first thread	0%	0%	0%	0%
Screw fracture—central threads	0%	0%	0%	0%
Screw fracture—most apical thread	0%	0%	0%	0%
Screw plastic deformation (no fracture)	90%	100%	90%	90%
Abutment misfit	90%	90%	90%	100%
Abutment shearing	60%	20%	0%	0%
Abutment loosening	50%	10%	0%	0%
Implant platform deformation	80%	100%	80%	70%
Implant external hexagon deformation	40%	50%	30%	60%
Implant internal hexagon deformation	0%	0%	0%	0%
There were no visible deformations	10%	0%	0%	0%

## Data Availability

Data are available upon request.
